# A Biomimetic Roll-Type Tactile Sensor Inspired by the Meissner Corpuscle for Enhanced Dynamic Performance

**DOI:** 10.3390/biomimetics10120817

**Published:** 2025-12-05

**Authors:** Kunio Shimada

**Affiliations:** Faculty of Symbiotic Systems Sciences, Fukushima University, 1 Kanayagawa, Fukushima 960-1296, Japan; shimadakun@sss.fukushima-u.ac.jp; Tel.: +81-24-548-5214

**Keywords:** tactile sensitivity, cutaneous receptors, Meissner corpuscle, biomimetics, rubber, electrolytic polymerization, hybrid fluid (HF), robotics

## Abstract

Highly sensitive bioinspired cutaneous receptors are essential for realistic human-robot interaction. This study presents a biomimetic tactile sensor morphologically modeled after the Meissner corpuscle, designed for high dynamic sensitivity achieved using a coiled configuration. Our proposed electrolytic polymerization technique with magnet-responsive hybrid fluid (HF) was employed to fabricate soft, elastic rubber sensors with embedded coiled electrodes. The coiled configuration, optimized by electrolytic polymerization, exhibited high responsiveness to dynamic motions including pressing, pinching, twisting, bending, and shearing. The mechanism of the haptic property was analyzed by electrochemical impedance spectroscopy (EIS), revealing that reactance variations define an equivalent electric circuit (EEC) whose resistance (*R_p_*), capacitance (*C_p_*), and inductance (*L_p_*) change with applied force; these changes correspond to mechanical deformation and the resulting variation in the sensor’s built-in voltage. The roll-type Meissner-inspired sensor demonstrated fast-adapting behavior and broadband vibratory sensitivity, indicating its potential for high-performance tactile and auditory sensing. These findings confirm the feasibility of electrolytically polymerized hybrid fluid rubber as a platform for next-generation bioinspired haptic interfaces.

## 1. Introduction

Current sensor fields are driving advances in healthcare and human-robotic or human-machine interaction for applications such as health management, disease screening, and rehabilitation feedback [1,2,3,4,5], and also various tactile engineering fields including industry, surgery, space, and mechanics [6,7,8,9,10,11,12,13,14,15,16]. In particular, haptic technology is significant for sensitive performance, providing automatic control and self-sustainability. The feasibility of obtaining haptic performance requires touch sensations corresponding to normal or shear forces, temperature, vibration, etc. In addition, the strategy of soft robotics is also crucial, utilizing soft materials involving elastomers, thin flexible sheets, rubber, etc.

Therefore, tactile technology has advanced electronic systems that simulate human skin through multifunctional structures that mimick cutaneous mechanoreceptors, including free nerve endings, Merkel’s disks, Krause end-bulbs, Meissner corpuscles, Ruffini endings, and Pacinian corpuscles. Biomimetic performance can be achieved by mimicking mechanoreceptors. And it also requires key features such as self-powered operation, flexibility, and stretchability to accommodate complex deformation, including significant elongation and bending, as well as a diverse and highly sensitive response to mechanical, thermal, and ionic stimuli. Thus a biomimetic strategy is indispensable. Artificially fabricated receptors result in a differences compared to other biosensors and strain gauges. Accordingly, our strategy involves using rubber as an essential material for the production of such biomimetic sensors and in order to reproduce receptor morphology via our proposed rubber-based electrolytic polymerization method, which satisfies these prerequisites.

However, the morphological strategy of biomimetic fabrication encounters many difficulties in terms of imitatively designing the configuration of mechanoreceptors, because engineering techniques are needed to morphologically realize the complex structure, such as a spiral or dendritic shape, capsule body, etc., under manageable production utilizing soft materials. For example, how the coil-like shape can be fabricated utilizing some rubber is a critical engineering problem. There is little research on the topic, except for Pacinian corpuscles [17,18] and Ruffini endings [19], in addition to our previous sequential studies. The morphological realization of the complex structure is very difficult; yet even then, current research has attempted to mimic morphological paradigms and systems. The present study addresses the development of a tractable technique for the fabrication of the complex configuration utilizing a soft rubber and deals with the structure of it being rolled up like a spring. Because the Meissner corpuscle, which has a roll-type configuration like a coil, is diagnostically elucidated, it is expected that the high tactility performance is a rapid response to the stimuli. we demonstrate a novel biomimetic sensor utilizing the morphologically modeled Meissner corpuscle. Thus, designs including other mechanoreceptors have been rarely reported in prior cutaneous receptor-based sensor studies.

In the present study, we focused on the sensitive performance of kinematic interfaces and diverse tactile sensations. By clarifying the response mechanism to various mechanical stimuli, the role-type structure is expected to enable good performance on variegated kinematic interfaces. According to our findings, its performance had highly sensitive voltage response characteristics of FA, defined by the EEC mechanism of a biomimetic sensor, and induced during the analysis of the EIS results.

## 2. Morphological Strategy

For the development of cutaneous mechanoreceptors in the biomimetic sensor, it is first necessary to understand the receptor properties, as shown in Table 1, which are categorized from the current literature summarizing anatomical findings [20,21,22,23,24]. Each receptor has distinct characteristics that can be exploited in specific biosensors with particular sensitivity functions. Regarding other sensory functions, although each receptor exhibits limited additional properties, some may also respond to ionic or mechanical stimuli, enabling applications in complex tactile and thermal sensing [25], and even gustation or olfaction [26]. The present study focuses on mechanical sensation. Reviewing the characteristics in Table 1, we adopted the Meissner corpuscle type of sensor because it appears suitable for sensing kinematic interactions, as indicated by its effective stimuli and sensory functions (shown in Table 1), including pressure, shear force, and deformation. Additionally, it may also provide high sensing performance for diverse tactile sensations for the following reasons: it has an FA with sufficiently high sensitivity, and its spatial acuity and receptive field are small, so that tactile perception of minute surface irregularities, such as small lumps, tumors, or bumps, can also be detected.

**Figure 1:**
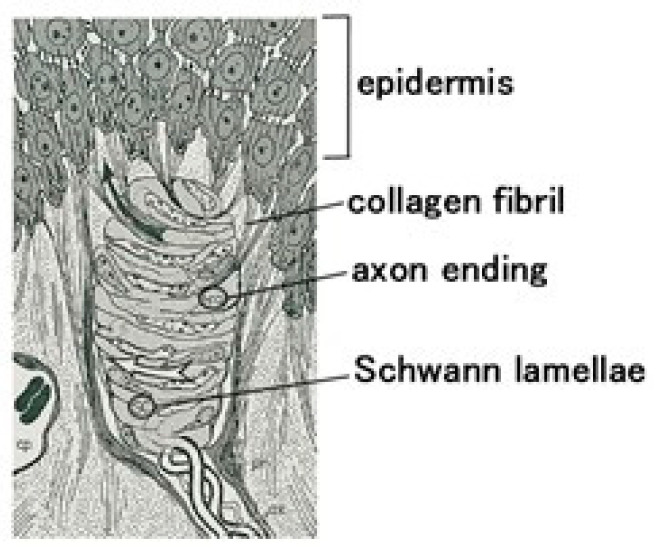
Diagnostic morphological structure of Meissner corpuscle [27].

**Figure 2:**
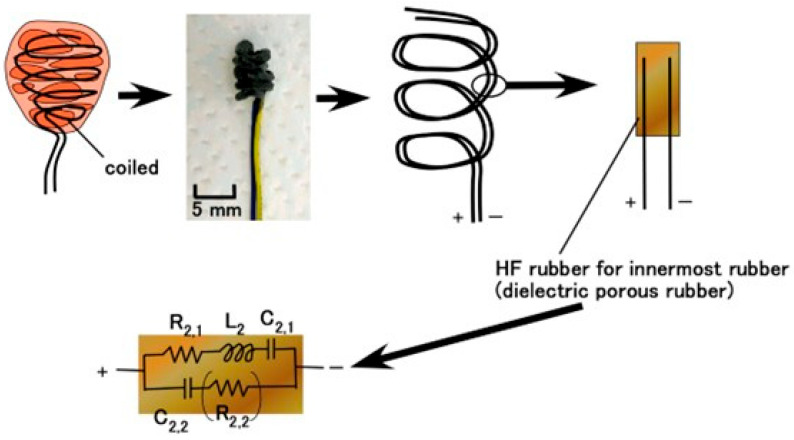
Biomimetic morphology of our proposed Meissner corpuscle and EEC.

While the diagnostic morphological structure of Meissner corpuscles is complex enough to be difficult to define precisely, as shown in Figure 1 [27], it can be mimicked by coiling electric wires, representing simulated nerves, in a spiral within the capsule [28,29]. However, biomimetic sensors employing such coiled structures have only recently been investigated. We propose the morphological structure shown in Figure 2. The rubber is solidified by our proposed electrolytic polymerization technique, as presented concisely below, and the rubber is adhered to the electric wires. Next, the rubber containing the electric wire is coiled like a spiral.

When a DC electric field is applied to the rubber, solidification occurs on the anode electrode and afterwards progresses toward the cathode [30]. Another prerequisite is compatibility among different types of rubbers—water-soluble rubbers such as NR and CR, and water-insoluble rubbers such as Q-rubber and U-rubber. Therefore, PVA was incorporated into these rubbers to enable mixing through emulsion polymerization. In addition, HF was adopted by using magnetic clusters fabricated from size-disparate metallic particles—10 nm Fe_3_O_4_ nanoparticles aggregated with 1 μm metallic particles (Ni or Fe)—under a magnetic field aligned with the electric field; the electric and thermal conductivities were enhanced along the clusters so that the sensitivity was improved. Using a metallic hydrate such as Na_2_WO_4_·2H_2_O allows for the rubber to become permeable and to be adhered to metals during electrolytic polymerization.

**Figure 3:**
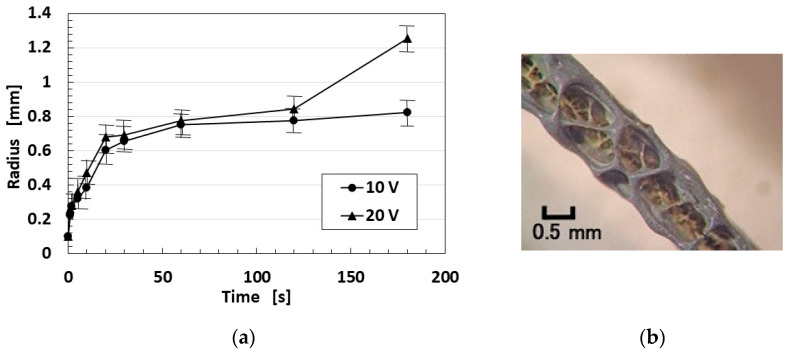
Electrolytically polymerized rubber: (**a**) incremental change in the thickness (radius) of the polymerized rubber under different applied voltages (10 V and 20 V); (**b**) microscopic image showing the porous surface of the electrolytically polymerized rubber formed on an electric wire (as shown in Figure 2).

Regarding the thickness of the electrolytically polymerized rubber on the electric wire, it varies with the applied electrochemical conditions, as indicated by “radius” which was measured by a vernier caliper with several samples per point in Figure 3a. The thickness of the coated rubber can be obtained by subtracting the 0.1 mm wire’s radius from the ordinate of Figure 3a. The larger the applied voltage, the thicker the electrolytically polymerized rubber layer formed. It is characteristic that, during the initial stage, the thickness increases exponentially. Therefore, even if the electrolytic polymerization period is very short (e.g., approximately 1 s), a measurable coating still develops. The surface of the electrolytically polymerized rubber is spongelike with many pores, as shown in Figure 3b. These pores are formed by the evolution and volatilization of H_2_ and O_2_ gases during the electrolysis of water in the electrolytic polymerization process.

By the application of the electric field, the isoprene molecules of CR and NR undergo crosslinking at the anode side, as represented by e in Equation (1), which was confirmed by our previous study using Raman spectroscopy and related analyses [31]. The isoprene acts as a specific monomer initiating cationic polymerization, thereby forming crosslinks [32,33]. Initially, the isoprene molecules of CR and NR (shown as a in Equation (1)) exist predominantly in the stable anionic state under latex conditions (b in Equation (1)) before forming radicals (c in Equation (1)). This radical isoprene functions as a cationic initiator for polymerization. Therefore, isoprene molecules become crosslinked into a polymer possessing an anionic character, as shown by e in Equation (1). Subsequently, the anionic crosslinked isoprene migrates toward the anode, enabling cationic polymerization; the electrons (d in Equation (1)) are then transferred to the anode. From the above-mentioned perspective of cationic polymerization occurring at the anode, the electric behavior between the electrodes of the fabricated sensor can be interpreted under the intrinsic condition that the electrolytically polymerized rubber exhibits anionic characteristics.
(1)
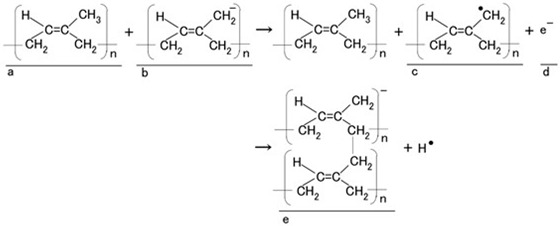


**Figure 4:**
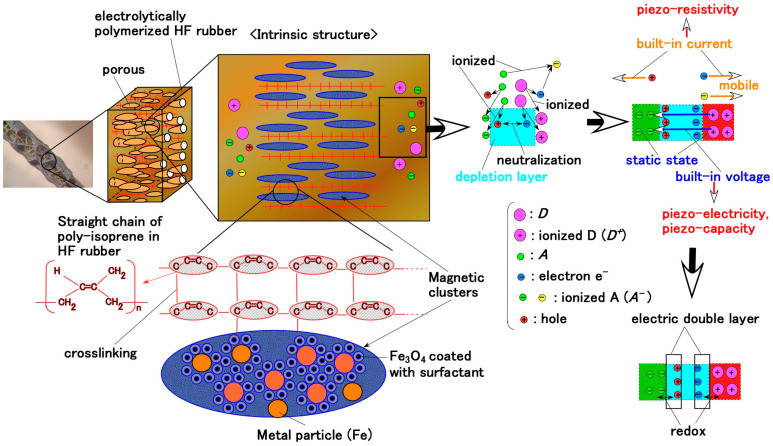
Physical model illustrating the fundamental behavior of electrons and ions within the HF rubber element.

**Figure A1:**
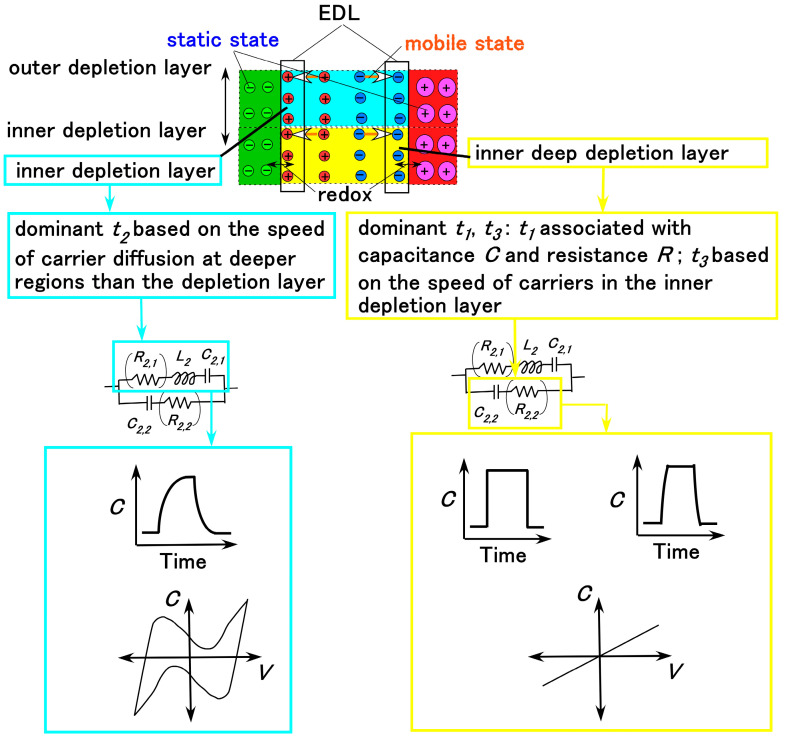
Physical model of dopant/acceptor species and EDL, and the relationship between EEC and changes in *C* and *C*–*V* profiles.

Regarding the EEC, as shown in Figure 2, to clarify the sensing mechanisms of the biomimetic sensor, the AC electrical characteristics were measured using an inductance/capacitance/resistance (LCR) meter. From the measured impedance data, we evaluated the electronic and ionic behavior in the EEC. In the present study, we compare the properties of our prior Meissner-, Krause end-bulb-, layered Ruffini endings-, and free nerve endings-type sensors [30]. These configurations are sandwiched structures with layers or concentrically cylinder-shaped using a few HF rubbers, and are not coil shaped. The EEC of the previous type coincides with that of the new type, as illustrated in Figure 2: the EEC consisted of a single equivalent unit comprising *R*_2,1_, *L*_2_, *C*_2,1_, and *C*_2,2_, with *R*_2,2_ being negligible. It is derived from the fundamental electron and ion behavior within the HF rubber element, as shown in Figure 4. The molecules of HF rubber and water, and the particles of Fe_3_O_4_ and Fe, serve as D and A species, respectively, which are ionized to D^+^ and A^−^, as the figure illustrates. When A^−^ is desorbed through hole transfer while D^+^ remains static, a built-in voltage is generated, giving the HF rubber piezoelectric characteristics. The mechanism of and the analytical findings regarding the creation of the built-in voltage were clarified in the previous research for the case of MCF [34,35]. MCF is the predecessor of HF, because HF was developed with easy production and reasonable ingredients in mind. The characteristics of MCF rubber are quantitatively and qualitatively the same as those of HF rubber. In addition, electrostatic energy can be stored between A^−^ and D^+^, imparting piezocapacitive behavior similar to that of a condenser. On the other hand, the holes, electrons, and A^−^ species that absorb electrons remain mobile, producing a built-in current and giving the HF rubber piezoresistive behavior. Holes approach negatively aggregated regions formed by A^−^ (desorbed by holes), while A^−^ species that absorb electrons migrate toward positively aggregated D^+^ regions, resulting in the formation of an EDL. The physical model proposing dopant/acceptor species and EDL formation was clarified in our previous research [36], the aim of which was the lighten stimulation of the MCF rubber corresponding a solar cell. The electrically charged carriers in the HF rubber under strain have the same performance as those under illumination. Typically, when the voltage is changed by any stimuli such as compression, strain, light, etc., over time, the time when the voltage reaches a constant value until exponentially rising or falling is defined by the time constant. The time constant is divided into three types *t_1_*, *t_2_*, and *t_3_* by the electric condition in the depletion layer of the material in which the particles or ions are dispersed. The time constant is relevant not only to the EEC but to the *C*–*V* profiles, which can be obtained a potentiostat, as shown in Figure A1 in Appendix A.

## 3. Materials

**Figure 5:**
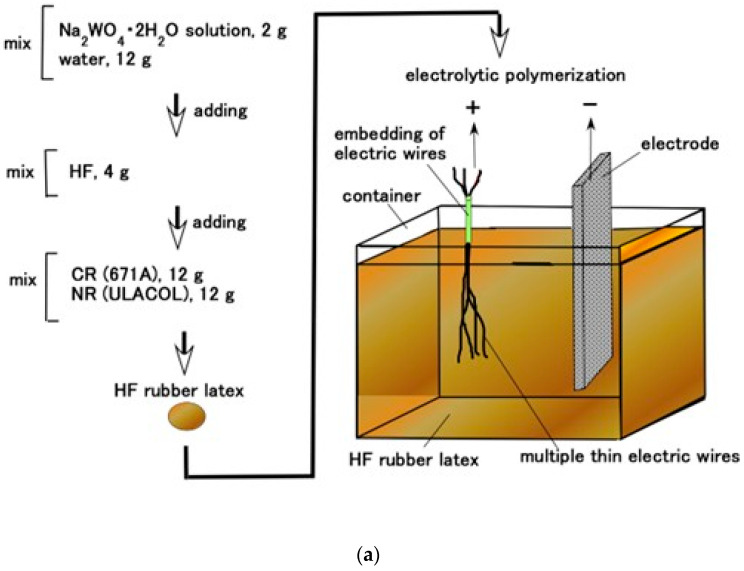

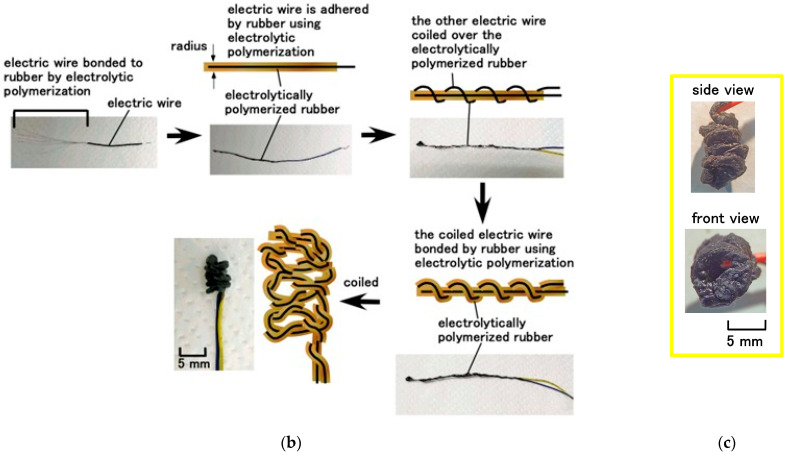
Morphological strategy of the biomimetic Meissner corpuscle: (**a**) electrolytic polymerization process; (**b**) fabrication sequence; and (**c**) images magnifying the consummated fabricate.

The 0.1 mm diameter electric wire was immersed in the HF rubber latex comprising Na_2_WO_4_·2H_2_O (Fujifilm Wako Chemical Co., Osaka, Japan), NR (ULACOL; Rejitex Co., Ltd., Atsugi, Japan), CR (671A; Showa Denko Co., Ltd., Tokyo, Japan), and HF, as shown in Figure 5a. The HF was composed of 3 g of Fe_3_O_4_ (Fujifilm Wako Chemicals Co., Ltd., Osaka, Japan), 3 g of Fe (M300 in about 50 μm particles; Kyowa Pure Chemical Co., Ltd., Tokyo, Japan), 3 g of water, 3 g of kerosene, 3 g of silicone oil (KF96, 1-cSt viscosity, polydimethylsiloxane; Shin-Etsu Chemical Co., Ltd., Tokyo, Japan), 21 g of PVA, and 4 g of sodium hexadecyl sulfate aqueous solution (C_16_H_33_NaO_4_S) as a surfactant (Fujifilm Wako Chemicals Co., Osaka, Japan). Mixing was conducted with 28 kHz ultrasonic stirring (UR-20P, Tomy Seiko Co. Ltd., Tokyo, Japan) in the present experiment. The mixing order is presented as shown in Figure 5a with a few seconds of stirring time. Regarding the production of the HF, the mixing order does not matter and the stirring time was a few hours. Incidentally, the production and measurement environments were at 45–50% humidity and at temperatures of 23–25 °C because these were the optimal conditions for the solidification of the rubber.

The electric wire was rendered an anode to be adhered to the rubber by electrolytic polymerization with 20 V, 2.7 A, and for 10 s (the applied electric field conditions were the same across experiments), as shown in Figure 5a, and then another electric wire was coiled around the electrolytically polymerized rubber, as shown in Figure 5b. The cathode of the electrode in the bath, as shown in Figure 5a, is a stainless-steel plate (18 mm width × 47 mm height × 1 mm thickness). And the distance between the electric wire and the cathode did not permit their touching in the bath (25 mm width × 60 mm length × 40 mm depth), which was at a temperature of about 15 °C. In the present experiment, two types of samples were prepared—one “with Ni & TiO_2_” and one “without Ni & TiO_2_”—by varying the compounding before the mixing of NR and CR, as shown in Figure 5a. The Ni was a carbonyl nickel powder (No. 123, Yamaishi Co., Ltd., Noda, Japan) with micron-sized, rough-surfaced particles, and the TiO_2_ (anatase type; Fujifilm Wako Chemical Co., Ltd., Osaka, Japan) served as an electron-transfer medium. Subsequently, the sample was immersed again in the HF rubber latex (Figure 5a), and a second electric field was applied with the coiled wire serving as the anode at 20 V, 2.7 A, and for 10 s. The final state of the electrolytically polymerized rubber, shown in Figure 5b, was a coiled structure. The coil had a pitch from 4 to 6 and was about 4 mm in diameter. Both the line-type (pre-coiling) and role-type samples were used in the experiments.

**Figure 6:**
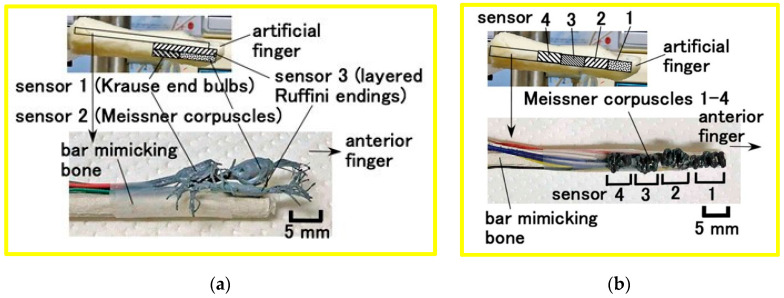
Artificial fingers incorporating biomimetic mechanoreceptors: (**a**) containing Krause end-bulbs, Meissner corpuscles, and layered Ruffini endings; (**b**) containing roll-type Meissner corpuscles.

The artificial biomimetic mechanoreceptors were embedded in U-rubber (Human Skin Gel; Exseal Co., Ltd., Gifu, Japan) mimicking a human finger, as shown in Figure 6. The finger was installed in the experimental apparatus. We fabricated two types of mechanoreceptors: (a) the previously developed types (Figure 6a) and (b) the newly proposed coiled Meissner-type receptor (Figure 6b). Each sensor was positioned as shown in the figures. For comparison, a finger containing a single mechanoreceptor was also used. These artificial fingers contain a core mimicking a human bone, fabricated from 8 mm diameter acrylic resin.

## 4. Experimental Procedure

**Figure 7:**
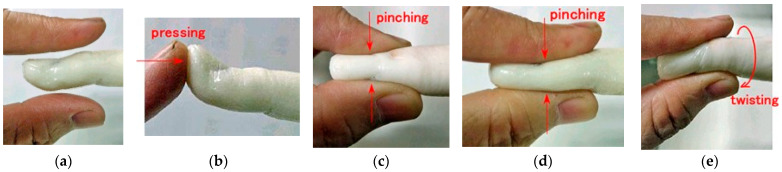
Physical deformation of a human finger under various motions: (**a**) no deformation; (**b**) pressing on a localized area; (**c**) transverse pinching; (**d**) vertical pinching; and (**e**) twisting.

We assumed various haptic situations corresponding to human body motions such as pressing, bending, pinching, and twisting, as shown in Figure 7. To investigate the haptic sensitivity induced by physical deformation under these diverse motions, several experimental setups were used.

**Figure 8:**
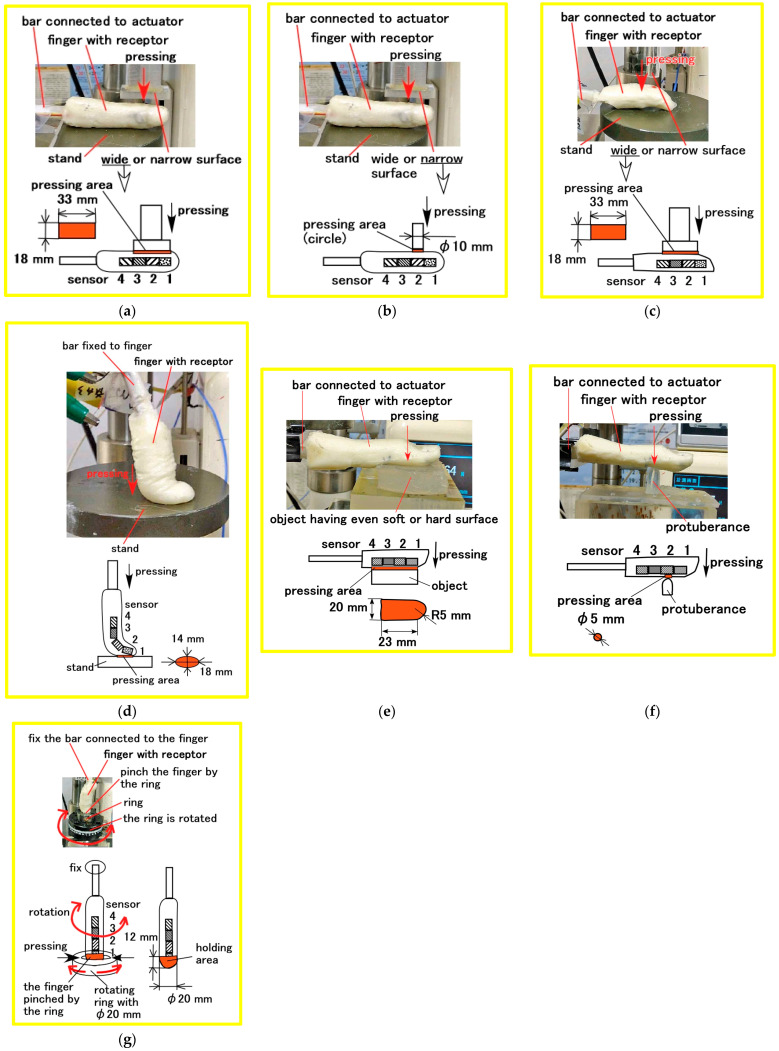
Specific physical motions applied to the artificial finger to clarify haptic sensitivity during experiments: (**a**) pressing on the wide surface of the flank to simulate lateral pinching, as in Figure 7c; (**b**) pressing on the narrow surface of the flank to simulate lateral pinching, as in Figure 7c; (**c**) vertical pinching on the wide surface to simulate pinching, as in Figure 7d, or pressing on a wide surface to simulate pressing, as in Figure 7b; (**d**) pressing longitudinally on the fingertip to simulate pressing, as in Figure 7b; (**e**) pressing on a broad area with either a soft or hard surface; (**f**) pressing on a localized protuberance; and (**g**) twisting by rotation to simulate twisting, as in Figure 7e.

**Figure 9:**
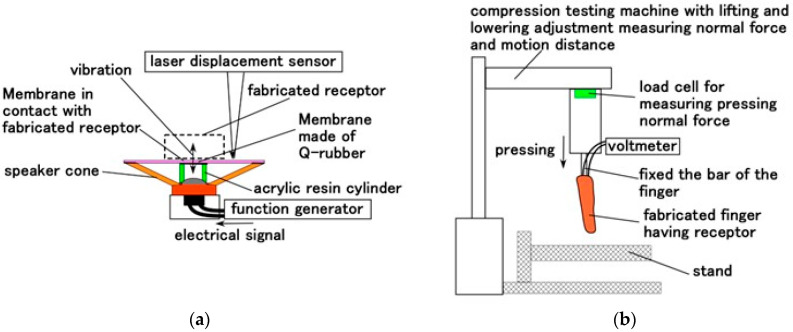
Mechanical visibility of the experimental apparatus: (**a**) for vibration sensitivity; (**b**) for haptic sensitivity by the dynamic motion, as in Figure 8.

As shown in Figure 8, the fabricated finger was subjected to pressing by a flat object or a protuberance, placed on a flat stand, or twisted. During pressing tests, the finger and object were fixed and moved using a compression testing machine (SL-6002; IMADA-SS Co., Ltd., Toyohashi, Japan) (Figure 9b). In the twisting test (Figure 8g), the fingertip was fixed and rotated. The voltage response from the receptor was measured using a digital voltmeter (PC710; Sanwa Electric Instrument Co., Ltd., Tokyo, Japan). It had an accuracy of 0.12%.

We also investigated vibration sensitivity using a vibration-inducing apparatus incorporating a speaker, as described in our previous study (Figure 9a). Based on the obtained frequency characteristics, the haptic sensitivity under the various motion conditions shown in Figure 7 was evaluated. The fabricated mechanoreceptors were placed in contact with a soft membrane (Young’s modulus: 1.8 MPa, made of Q-rubber which was diluted by using thinner because it has large elasticity) adhered to the edge of an acrylic resin cylinder attached to the speaker cone. Vibrations were applied to the membrane by electrical signals from a function generator in the speaker, and the resulting displacement was measured using a laser displacement sensor.

**Figure 10:**
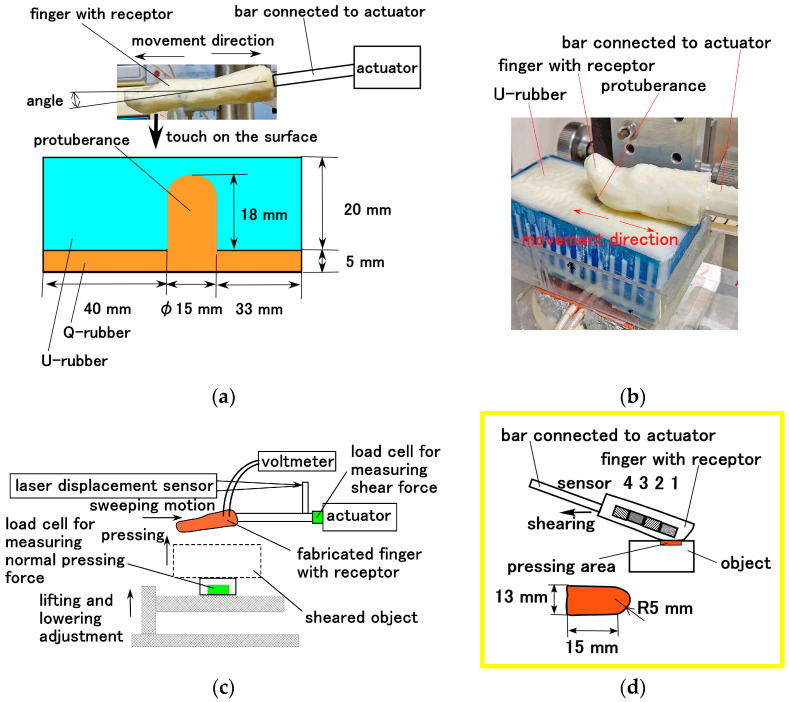
Experimental apparatus for evaluating haptic sensitivity under shearing motion: (**a**) schematic of the positional relationship between the fabricated finger and the sheared object; (**b**) photograph showing the positional relationship; (**c**) experimental setup of the shearing test instrument; and (**d**) schematic sensor placement within the finger under shearing.

In addition, shear motion was examined using the apparatus shown in Figure 10. The fabricated finger was sheared by an actuator against a Q-rubber object with a protuberance. The Q-rubber (40 mm width × 80 mm length × 30 mm depth) had 1.56-μm *R_a_*, 4.8-μm *R_z_*, and 1.83-μm *R_q_* surface roughness, and its surface was sleek and soft with a 2.3 coefficient of dynamic friction. The protuberance had a diameter of 8 mm and was made of stainless-steel which was immersed in the Q-rubber (Figure 10a). The finger was swept at a 5 mm/s controlled speed and contact force. The contact force was also measured as the force vertically on the Q-rubber surface, because it was changed under shearing owing to the deformation of the elastic Q-rubber, and then the initial normal force was set to 1.5 N.

To clarify the sensitivity mechanisms of the fabricated sensors, the AC electrical properties were measured using an LCR meter (IM3536; Hioki Co., Ltd., Ueda, Japan). It had an accuracy of 0.05%, a 0.1 msec response time, a 1 V excitation amplitude, a 4 Hz–8 MHz frequency response range, and a maximum of 64 repeats.

## 5. Results and Discussion

### 5.1. Vibration Sensitivity

**Figure 11:**
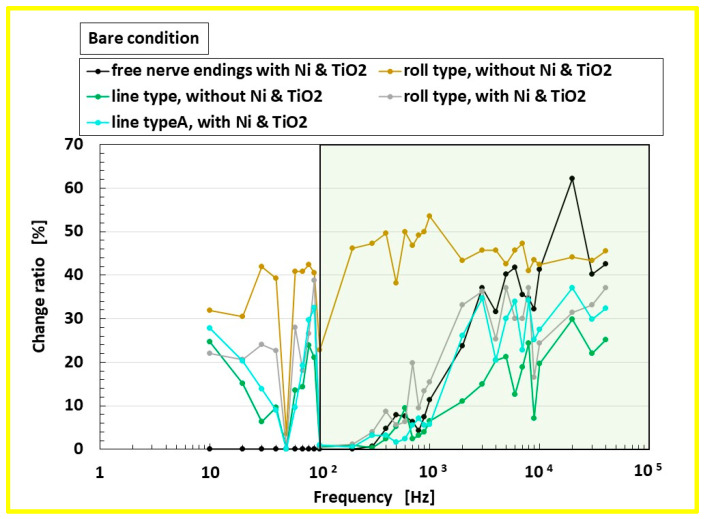
The vibration sensitivity of the fabricated mechanoreceptors in the experiment shown in Figure 9a, comparing conditions with and without Ni and TiO_2_, and between roll-type and line-type Meissner corpuscles, as shown in Figure 5.

Figure 11 shows the ratio Δ*V*/*V*_0_ of the power spectrum (FFT windowing: hamming, FFT length: 2048 points) of the receptor changing voltage Δ*V* to that of the measured voltage of the applied vibration’s amplitude on the soft membrane *V*_0_, obtained from the experiment shown in Figure 9a. The measurement was conducted under a bare-sensor condition, without U-rubber covering the receptor. The novel Meissner-type mechanoreceptor exhibits a greater response ratio in the low-frequency range (below approximately 100 Hz, indicated by the pale green zone in the figure) compared with free nerve ending sensors. It was clarified that other mechanoreceptors, except for the free nerve ending sensors, show lower response ratios in the lower frequency range, as demonstrated in our previous auditory sensitivity study.

In addition, in the case of the roll-shaped Meissner-type receptor fabricated without Ni and TiO_2_, the response ratio is higher across both low- and high-frequency regions. The complex motions of the artificial finger with the receptor (Figure 7) thus exhibit vibration sensitivity across a wide frequency range. Therefore, the results obtained from Figure 11 indicate that the optimal configuration of the biomimetic mechanoreceptor is the roll-type Meissner corpuscle fabricated without Ni and TiO_2_, which offers the best performance.

On the other hand, regarding the vibration sensitivity of the roll-type Meissner corpuscle without Ni and TiO_2_, this receptor is responsive across a wide frequency range, which contrasts with the characteristic low-frequency sensitivity of Meissner corpuscles. In addition, the auditory sensitivity of this biomimetic receptor also extends over a wide frequency range, in contrast to our previously proposed mechanoreceptors, such as free nerve endings (other mechanoreceptors have the same tendency of sensitivity to frequencies lower than 100 Hz, as shown in the figure), which was primarily sensitive to frequencies above approximately 100 Hz (the pale green area in Figure 11). In conclusion, our proposed roll-type receptor is suitable for applications requiring vibration sensitivity across a broad frequency range.

### 5.2. EIS and EEC

**Figure 12:**
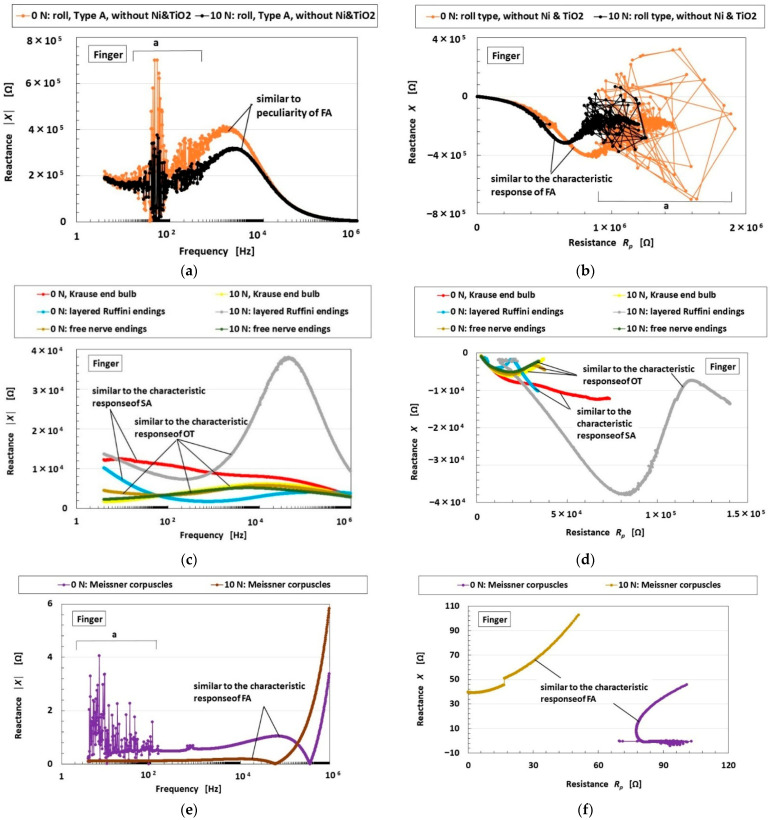

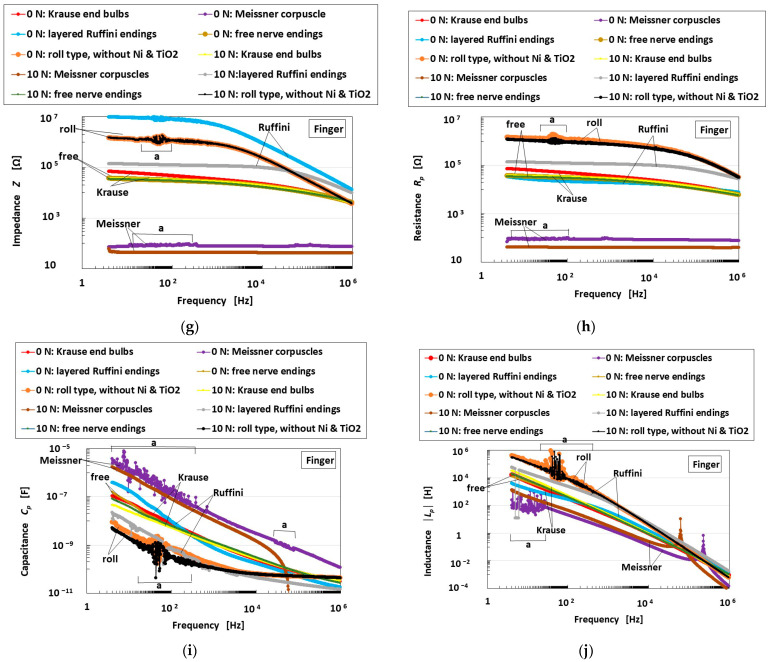
EIS results for our proposed roll-type Meissner corpuscles (designated as the “roll-type” sensor) compared with our previously proposed mechanoreceptors: (**a**,**c**,**e**) show the relationship between reactance and resistance; (**b**,**d**,**f**) show absolute reactance; (**g**) impedance; (**h**) resistance; (**i**) capacitance; and (**j**) absolute inductance.

**Figure 13:**
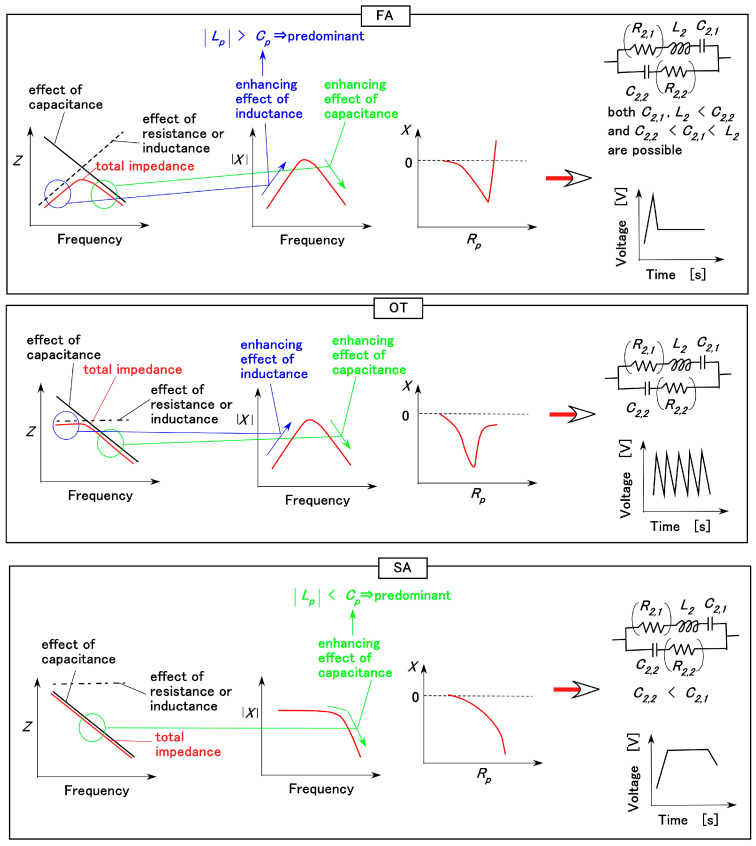
Relationships among impedance, absolute reactance, and the reactance-resistance relationship obtained by EIS, together with corresponding EEC and voltage responses for the FA, OT, and SA types [37].

The sensitivity to the various motions of the finger with the biomimetic mechanoreceptor, as shown in Figure 7, stems from the EEC of the receptor, which induces the FA, SA, and voltage responses. The EEC can be inferred from EIS measurements, which were obtained as shown in Figure 12, under the condition that the receptor was enveloped by the finger’s U-rubber layer. The relationships among the FA, SA, voltage responses, and the EEC are shown in Figure 13, as described in our previous study [37]. They are derived from changes in absolute reactance and the relationship between reactance and resistance. The firing rate is generally classified as either SA or FA to the applied stimulus; and FA is characterized by a voltage peak, while in SA voltage changes occur smoothly. However, the classification can be extended to include another type (OT), which is also characterized by fluctuating voltage changes, as shown in Figure 13. The resistances *R*_2,1_ and *R*_2,2_ shown in parentheses in the EEC diagram in Figure 13 are negligible under some conditions. The results obtained when the applied force was maintained using the experimental apparatus shown in Figure 9b, with the applied force held as in Figure 8d, were also compared.

The roll-type Meissner corpuscles without Ni and TiO_2_, the sensor proposed in this study, exhibit FA characteristics that differ from those of other mechanoreceptors showing SA or OT behavior, as shown in Figure 12c,d, and display dominance of *L_p_* over *C_p_* as shown in Figure 12g–j. This can be summarized as follows:

FA: Roll-type Meissner corpuscles (0 N, 10 N),
  previous type Meissner corpuscles (0 N, 10 N);

OT: Krause end-bulbs (10 N), layered-type Ruffini endings (10 N),
  free nerve endings (0 N, 10 N);

SA: Krause end-bulbs (0 N), layered-type Ruffini endings (0 N).

The results of EIS for other receptors are the same as those obtained under the applied-force conditions. From the sensitivity paradigm shown in Figure 13, our proposed sensor demonstrates high performance and strong sensitivity to mechanical motion and deformation.

Incidentally, the disturbance observed in the roll-type Meissner corpuscles, as well as the other mechanoreceptors, indicated by “a” in Figure 12a,b and partly by *Z*, *R_p_*, *C_p_*, and *L_p_* in Figure 12c–j, is predominantly due to the following causes: (1) the perturbed behavior of HF rubber and water molecules, and of Fe_3_O_4_ and Fe particles; (2) the mobile electrons passing through non-conductive materials such as rubber or surfactant via a tunneling effect, a phenomenon we have clarified theoretically; and (3) impurities introduced during the sensor fabrication process. In particular, the complex behavior of the particles and ions in the HF rubber is based on the tunnel effect, as presented in our previous research [36], so that the disturbance exists. This mechanism may be investigated further.

From the results of Figure 11 and Figure 12, we present the composition of a biomimetic mechanoreceptor sensor in terms of its vibro-tactility, as shown in Table 2. Our proposed roll-type Meissner corpuscle is sensitive over a wider frequency range than any other sensor.

### 5.3. Mechanical Response

**Figure 14:**
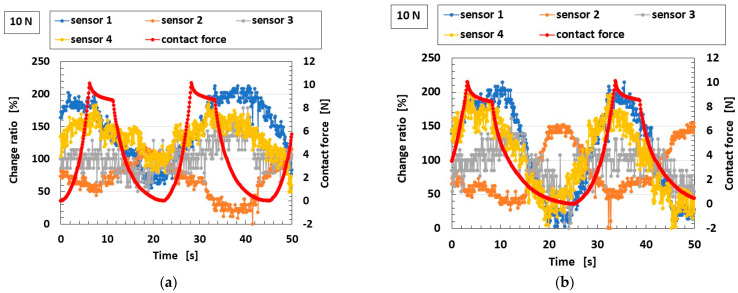

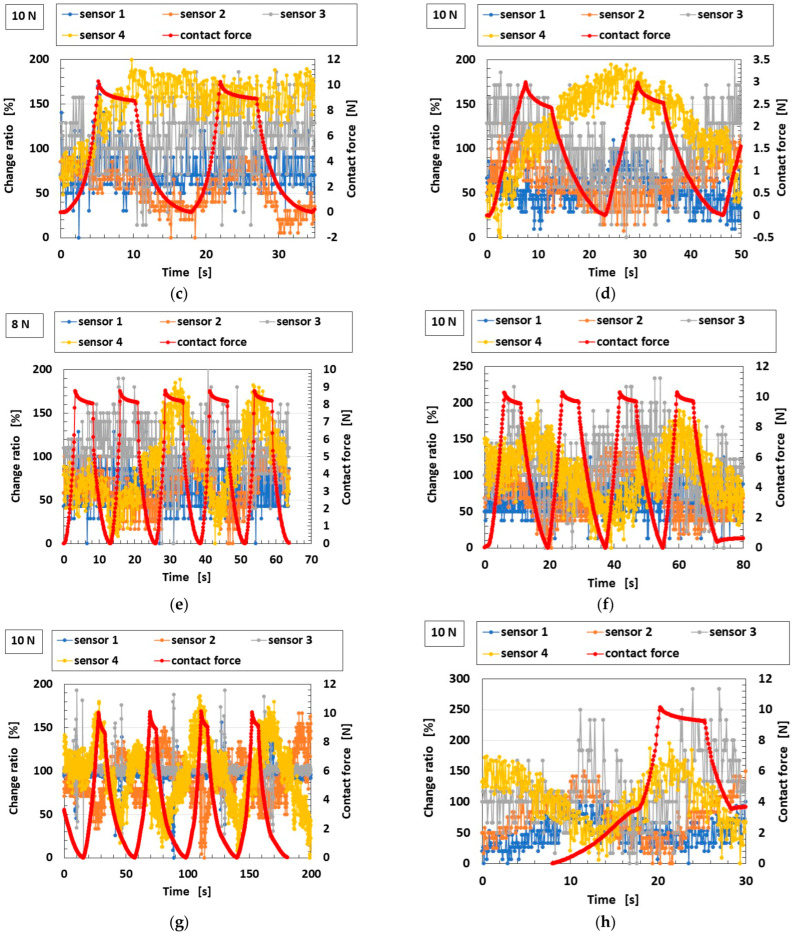

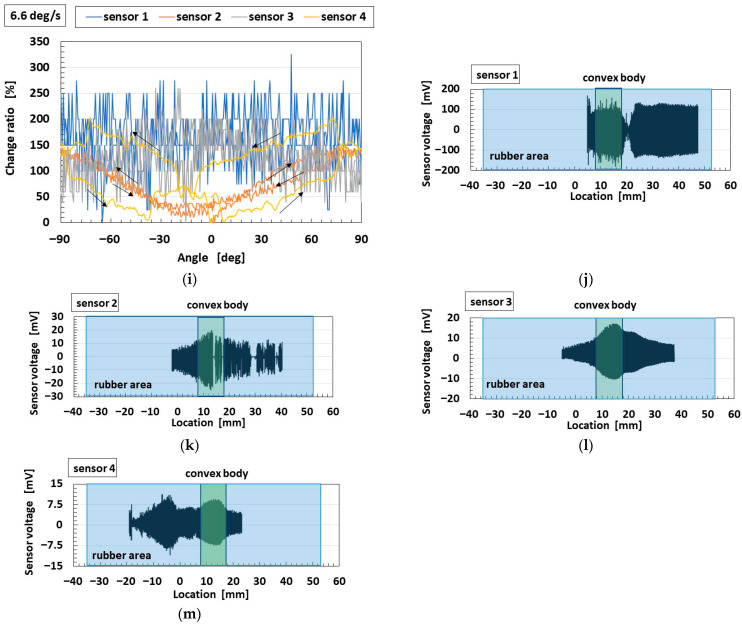
Sensitivity of the proposed roll-type Meissner corpuscles (without Ni and TiO_2_) to various finger motions, measured using the experimental setups shown in Figure 8, Figure 9 and Figure 10 and the finger model in Figure 6b; (**a**) pressing on wide lateral surface, as in Figure 8a; (**b**) pressing on a narrow lateral surface, as in Figure 8b; (**c**) pinching on a wide surface, as in Figure 8c; (**d**) pressing longitudinally on the fingertip, as in Figure 8d; (**e**) pressing on a broad area of a hard surface, as in Figure 8e; (**f**) pressing on a broad area of a soft surface, as in Figure 8e; (**g**) pressing on a hard Q-rubber protuberance, as in Figure 8f; (**h**) pressing on a soft Q-rubber protuberance, as in Figure 8f; (**i**) twisting test: fingertip fixed and rotated, simulating torsional motion, as in Figure 8g; and (**j**–**m**) shearing tests: lateral movement across surfaces with different textures, as shown in Figure 10.

**Figure 15:**
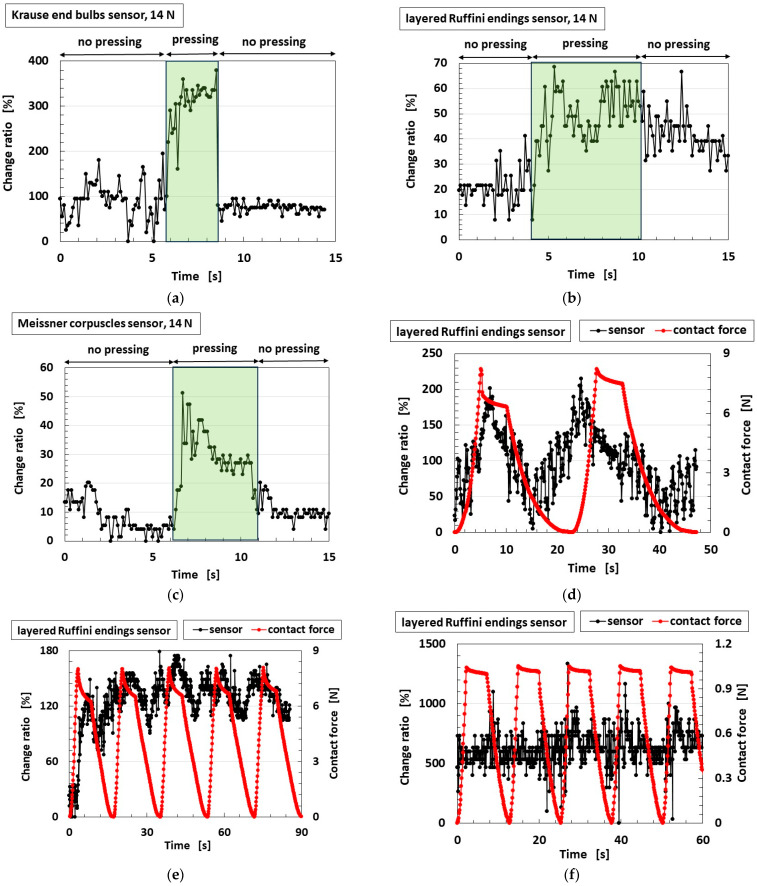

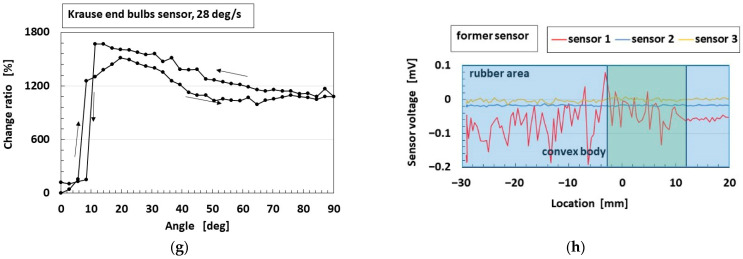
Sensitivity to the various motions of the finger involving the previously proposed biomimetic mechanoreceptor measured using the experimental setups shown in Figure 8, Figure 9 and Figure 10 and corresponding to the results for the proposed roll-type Meissner corpuscles (Figure 14): (**a**–**f**) Finger equipped with a single receptor type: (**a**) lateral pinch, as in Figure 8a, corresponding to Figure 14a; (**b**) wide press on a rigid surface, as in Figure 8c, corresponding to Figure 14c; (**c**) pressing on a hard, flat floor surface, as in Figure 8d, corresponding to Figure 14d; (**d**) pressing on a soft, flat Q-rubber plate, as in Figure 8e, corresponding to Figure 14f; (**e**) pressing on a hard Q-rubber protuberance, as in Figure 8f, corresponding to Figure 14g; (**f**) pressing on a soft Q-rubber protuberance, as in Figure 8f, corresponding to Figure 14h; (**g**) finger equipped with three types of receptors, as shown in Figure 6a, twisted by rotation, as in Figure 8g, corresponding to Figure 14i; and (**h**) shearing test, corresponding to Figure 14j–m.

The measured results showing the sensitivity of the biomimetic mechanoreceptor to various finger motions, obtained from the experimental setups shown in Figure 8, Figure 9 and Figure 10, are presented in Figure 14 for the proposed roll-type Meissner corpuscles without Ni and TiO_2_ (using the configuration in Figure 6b) and in Figure 15 for the previously proposed mechanoreceptors (using the configuration in Figure 6a). The ordinates in the figures denote the ratio Δ*V*/*V*_0_ of the voltage changing by strain Δ*V* to the initial voltage *V*_0_. In some cases, the contact force was repeated. The voltage of the sensor initially exhibits a built-in voltage relative to this initial value, which is critical. Therefore, the ordinate in Figure 14a–i presents the ratio of voltage change relative to the initial state.

Regarding our proposed roll-type Meissner corpuscles, according to the performance of the finger’s deformation by the varied motions, the location of the sensor that most strongly responds to the applied force, and where the voltage change is large or delayed, differs among motion types. This is attributable to differences in stress concentration arising from the various modes of motion. In any case, our proposed sensors are sensitive. As for shearing motion, all sensors also show strong sensitivity to the convex body (delineated in pale green in Figure 14j–m) within the U-rubber matrix (delineated in pale blue in Figure 14j–m), as shown in Figure 10.

**Figure 16:**
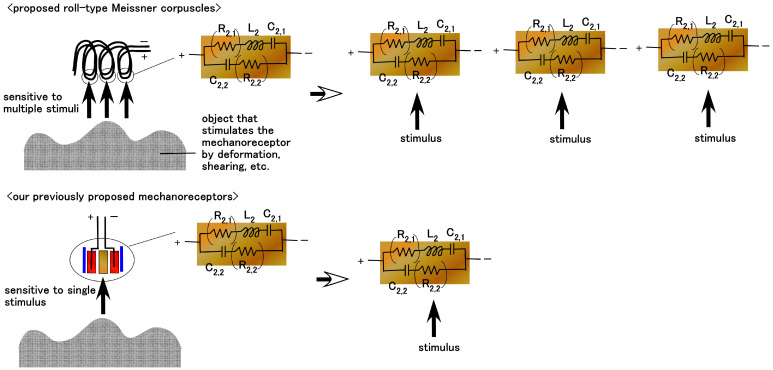
Schematic diagram showing the difference in sensitivity between our proposed roll-type Meissner corpuscles and our previously proposed mechanoreceptors.

Compared with our proposed roll-type Meissner corpuscles, the quantitative sensitivity of the previous types is smaller than that of the roll-type in the case of pressing on a wide rigid surface; a hard, flat, large floor surface, or a hard Q-rubber protuberance, as shown in Figure 15b,c,e. In the case of shearing, as shown in Figure 15h, this is particularly evident. These results can be explained as follows. As shown in Figure 16, while the previously proposed mechanoreceptors exhibit changes in one pair of built-in voltages, as shown in Figure 4, which defines one pair of EECs structured by *L*_2,1_, C_2,1_, and *C*_2,2_, as shown in Figure 13, by a single stimulus, our proposed roll-type Meissner corpuscles exhibit changes in multiple pairs of built-in voltages created by the spring-like shape under multiple stimuli. In addition, the structure of the coils, such as our proposed roll-type Meissner corpuscles, can be deformed more than that of the sandwiched-layer type used in the previously proposed mechanoreceptors, so that it is more sensitive to varied motion. Consequently, the effect of coil fabrication on sensitivity enhancement is significant.

The errors in Figure 14 and Figure 15, as well as the one in Figure 11, depend on the accuracy of the measurement instruments used, which were less than 1%. In general, SD connotes the measurement errors stemming from both the equipment’s accuracy and the measurer’s measurement technique. Therefore, it is about a few % points. In contrast, the perturbations in the figures seem to be errors, but are not correct. Because they are due to the phenomena of the HF rubber in which countless particles and ions are dispersed. The perturbation, as shown in the figures, can be considered to be due to the foregoing complex behavior of the particles and ions in the HF rubber, which is based on the tunnel effect. Regarding Figure 14 and Figure 15, another cause of the perturbations can also be considered as follows. The behavior of the particles and ions is changed by the strain, and the behavior is different according to the location of sensors 1–4 because the strain is different at different locations on the strained finger. For example, because sensor 1 in the case of twisting, as shown in Figure 14i, is pinched to be held by its base, the change ratio of sensor 1 remains almost constant, and the perturbation stems from the strain at the held base, and is not a measurement error.

## 6. Conclusions

The role-type structure mimicking the Meissner corpuscles is a critical strategy for enhancing the sensitivity of artificially fabricated mechanoreceptors to dynamic deformation in an artificial human finger by various motions, such as pressing, pinching, twisting, bending, and shearing. The coiled morphology responds to multiple stimuli arising from finger deformation, producing a highly sensitive voltage response characteristic of FA, as evidenced by the EIS results that define the EEC mechanism of the biomimetic sensor. In addition, the coil’s flexibility yields high performance in response to dynamic movement, resulting in high sensor sensitivity. In particular, our proposed role-type sensor demonstrates high quantitative sensitivity when pressing on a wide, flat, rigid surface or on a hard protuberance, and during shearing. Our proposed role-type sensor also features vibratory sensitivity over a range from low to high frequencies. Consequently, our results for the morphologically mimicked fabrication coincide with the well-known diagnostic characteristics of the Meissner corpuscles, namely FA and low-frequency vibratory sensitivity. Our proposed roll-type receptor is also suitable for applications involving auditory sensitivity over a wide frequency range.

Thus, the findings elucidated in the present research are relevant to mechanical performance, specifically to the haptic sense from among the five senses (tactile, olfactory, gustatory, auditory, and visual sensations). From another aspect of the performance of the biomimetic sensor, ionic functions such as olfaction and gustation may also be feasible in the same sensor. This is because the sensitivity of all five senses arises from the behavior of electrons and ions within the sensor material. Therefore, it is expected that the role-type sensor mimicking Meissner corpuscles will also demonstrate ionic performance. In addition, thermo-sensitivity in our proposed sensor remains to be clarified. In future studies, we plan to investigate and elucidate these additional properties, including gustatory function.

## Figures and Tables

**Table 1:** Characteristics of cutaneous mechanoreceptors in human skin summarized in the literature [20,21,22,23,24].

	Meissner Corpuscles	Pacinian Corpuscles	Merkel’s Disk	Ruffini Endings	Free Nerve Endings	Krause End-Bulbs
Type	FAI (RAI)	FAII (RAII)	SAI	SAII	-	-
Adaptation rate	fast	fast	slow	slow	-	slow
Stimulus frequency [Hz]	3–40	40–500+	0.4–3	100–500+	-	40–80
Spatial acuity [mm]	3–4	10+	0.5	7+	-	-
Receptive field [mm]	3–5 small	>20 very large	2–3 small	10–15 large	-	-
Conduction velocity [m/s]	35–70	30–90	16–96	20–100	-	-
Effective stimuli	temporal changes in skin deformation	temporal changes in skin deformation	spatial deformation, sustained pressure, curvature, edge, corners	sustained downward pressure, lateral skin stretch, skin slip	pain, temperature, crude touch	cold temperature, light touch
Sensory function	low-frequency vibration and motion detection, tactile flow (slip) perception, light touch perception	high-frequency vibration detection	pattern/form detection, texture perception, tactile flow perception	finger position, stable grasp, tangential force, motion direction	nociception, thermoreception, general touch	thermoreception, localized touch in specific areas

**Table 2:** Composition of biomimetic mechanoreceptor sensor in terms of vibro-tactility.

Biomimetic Mechanoreceptor	Sensitivity Frequency Range
Our proposing roll-type Meissner corpuscles	10–40,000
Our previously proposed mechanoreceptors (Krause end-bulbs, layered Ruffini endings, free nerve endings, Meissner corpuscles, and layered Pacinian corpuscles) [38]	100–40,000
Other research on Pacinian corpuscles [17]	10–300
Other research on Pacinian corpuscles [18]	20–1000
Other research on Ruffini endings [19]	1–40

## Data Availability

The original contributions presented in this study are included in the article. Further inquiries can be directed to the corresponding author.

## Nomenclature

A	acceptor
*C*	current
CR	chloroprene rubber
D	dopant
EDL	electrical double layer
EEC	equivalent electric circuit
EIS	electrochemical impedance spectroscopy
FA	fast adaptation
Fe_3_O_4_	magnetite
HF	hybrid fluid
MCF	magnetic compound fluid
NR	natural rubber
PVA	polyvinyl alcohol
Q-rubber	silicone rubber
RA	rapid adaption
SA	slow adaptation
SD	standard deviation
U-rubber	urethane rubber
*V*	voltage

## Appendix A

The physical model proposing dopant/acceptor species and EDL formation as shown in Figure 4, and the relationship between EEC and changes in *C* and *C*–*V* profiles are shown in Figure A1.
